# The Synergistic Effect of Bleomycin, Triamcinolone and Epinephrine in Treatment of Hemangioma and Arteriovenous Malformations

**Published:** 2012-07

**Authors:** Hamid Masiha, Heydar Ali Nikpour, Mohammad Esmaeil Hasani, Abolhasan Emami, Mehryar Jafari, Ali Manafi

**Affiliations:** 1 Private practis Metairie, LA, USA.; 2 Department of Plastic Surgery, Shiraz University of Medical Sciences Shiraz, Iran; 3 Department of Plastic Surgery, Tehran University of Medical Sciences, Tehran, Iran; 4 Medical Student, Tehran University of Medical Sciences, Tehran, Iran

**Keywords:** Bleomycin, Triamcinolone, Epinephrine, Hemangioma, Arteriovenous malformation

## Abstract

**BACKGROUND:**

Hemangioma is the most common tumor of neonatal period and it is almost always appeared by the end of the first week of life and can be found mostly in head and neck area. This study evaluated the synergistic effect of bleomycin, triamcinolone and epinephrine in treatment of hemangioma and arteriovenous malformations.

**METHODS:**

In this multicenter study, a combination of bleomycin, triamcinolone and epinephrine was injected intralesionaly for treatment of hemangiomas and arteriovenous malformations and their synergistic effect was evaluated in 32 patients.

**RESULTS:**

Hemangiomas and low-flow arteriovenous malformations were treated well with their combination while port-wine spots and high-flow lesions response were relatively poor.

**CONCLUSION:**

It seems that the combination of bleomycin, triamcinolone and epinephrine may be a good choice in treatment of hemangioma and arteriovenous malformations.

## INTRODUCTION

In 1982, Mulliken and Glowacki introduced a classification for vascular birth marks and divided these lesions into two main categories: hemangiomas and vascular malformations.^1^ The term hemangioma asserts a distinct group of proliferative lesions of childhood which is more likely to be a tumor rather than a vascular malformation.^2^ Hemangioma is the most common tumor of neonatal period which occurs in 1-2.6% of newborns.^3^ It is a subcutaneous red-bluish mass with a strawberry-like surface and shows a female gender tendency (F:M=3:1).^4^,^5^ It is almost always present by the end of the first week of life and can be found in head and neck (60%), body trunk (25%) and extremities (15%).^6^ There are two types of hemangiomas: Congenital and infantile. Congenital hemangiomas are rapid involution congenital hemangiomas (RICH) and non-involution congenital hemangiomas (NICH). The RICH hemangiomas are present at birth and tend to regress during few months while NICH hemangiomas do not involute during time and need to be treated with surgery during childhood. The infantile hemangioma is a benign neoplasm with three discrete phases including proliferative, involution and involuted phase. This type of hemangioma is not apparent at birth and has two rapid proliferation phases at 1-2 month and 4-5 months after birth. Its growth can be exponential and unpredictable.^7^

Vascular malformations are developmental vascular deformities that are originated from anomalies of embryonic vascular system between 4th and 10th week of fetus life. Vascular malformations are subdivided into high-flow and low-flow. High-flow malformations include arteriovenous malformations and low-flow category includes capillary, venous, lymphatic and combined malformations.^8^ They have bluish, compressible clinical view and are located commonly in head and neck. They are present at birth and unlike hemangiomas, they do not exhibit episodes of growth and involution. They are not symptomatic, although there have been cases with swelling or pain complaint.^7^

There are some treatment options for both hemangiomas and vascular malformations. Treatment of hemangioma depends on its site and phase. Flash lamp pumped dye laser or Nd:YAG laser therapy, imiquimod, systemic therapy with steroids or β-blockers, oral corticosteroids, pingyanmycin (bleomycin A5), alpha-interferon and surgery are treatment modalities for hemangiomas.^7^ Treatment of venous malformations could be more complicated. A variety of treatment options are applied by physicians including surgery, sclerotherapy, irradiation, electrocuagulation, cryotherapy, laser and compression.^9^

Bleomycin A5 is a sclerosing agent that affects vascular endothelium. It is administrated intralesionaly and has shown a success rate of over 90%.^10^ Its easy usage and safety beside effectiveness made it an ideal therapeutic modality in complicated vascular lesions^11^ and those lesions which did not respond to steroids and/or laser therapy.^7^ In this study, we have used bleomycin, triamcinolone and epinephrine as a composition to treat hemangiomas and arteriovenous malformation in 32 patients during a 4 years period.

## MATERIALS AND METHODS

A group of 32 patients entered our multicenter study (9 males and 23 females). The age range (7 months-52 year) (median: 22.6 years). Six participants suffered from hemangioma and four had port wine stains while the other twenty two lesions were mixed lesions including arteriovenous malformation (two high-flow arteriovenous malformation and the rest were capillary and venous malformation). Size of lesions varied from 1.5 cm to those lesions that covered almost half of patient’s face or in some cases extended to neck area. In cases other than hemangiomas, including high flow arteriovenous malformation and mixed lesions angiography by using Digital Subtraction Angiography (DSA) and in venous lesions MR venography was performed.

Each patient’s CBC, FBS and BUN were checked before injection. The maximum injections were 6 injections (one injection per month). The injection composition was bleomycin, triamcinolone and epinephrine. The bleomycin mean dose was 2.5-3 mg in major lesions, it was 0.25-0.3 mg per kilogram of body weight. Triamcinolone amount was 2.5-15 mg per day for adults and in children between 6 to 12 years old and was 2.5-8 mg per day for children under 6 years. Epinephrine dosage was 1/100000 of each injection mixture. The injection was performed intralesionally. Anesthesia method for most of the patients was general anesthesia or deep sedation and we conducted local anesthesia for small lesions. The mean follow-up duration was 1.3 years with a range of 4 months to 4 years.

In order to reduce the risk of atrophy or necrosis, in cases that showed proper improvements, after first and second injections, the interval between second and third injections were prolonged to 2 or 3 months instead of only one month. In case of progressive shrinkage of the lesions, we postponed the next injection for six months and repeated the injection only if the lesion stopped regressing and still seemed to need booster injection.

## RESULTS

**Fig. 1:**
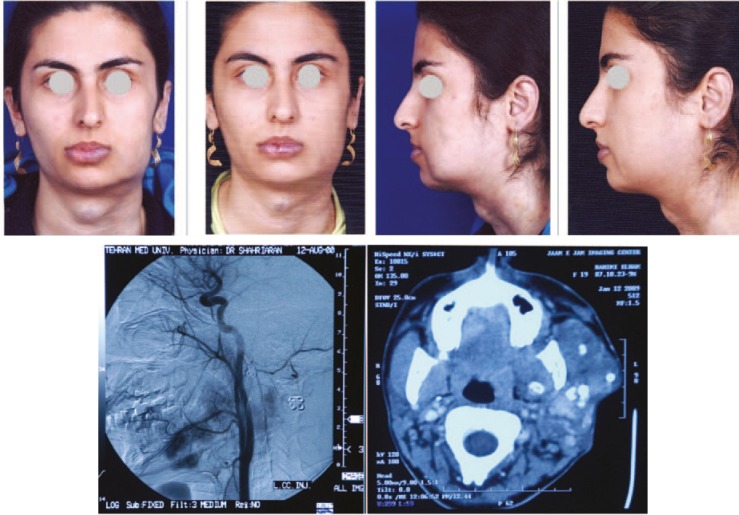
A 30 years old female patient with venous malformation of lower lip, face, neck. She has had 6 injections of combination (bleomycin, triamcinolone and epinephrine), 3 injections with 1 month intervals and 3 injections with 2 months intervals. Tumor blush in arterial phase of DSA is evident and CT Scan demonstrates soft tissue edema and phlebolithes.

**Fig. 2:**
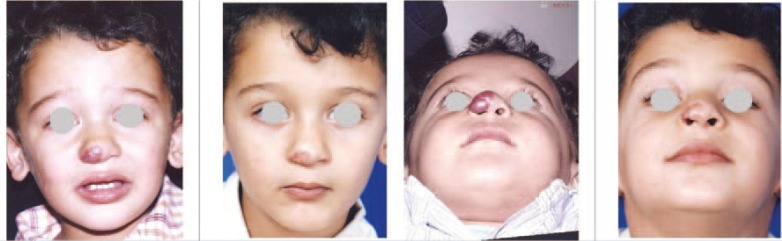
A 3 years old boy with nasal tip straw berry cap malformation (left). The patient received 2 injections of the combination of bleomycin, triamcinolone and epinephrine with 3 months interval. The outcome after 1 year (Right).

**Fig. 3:**
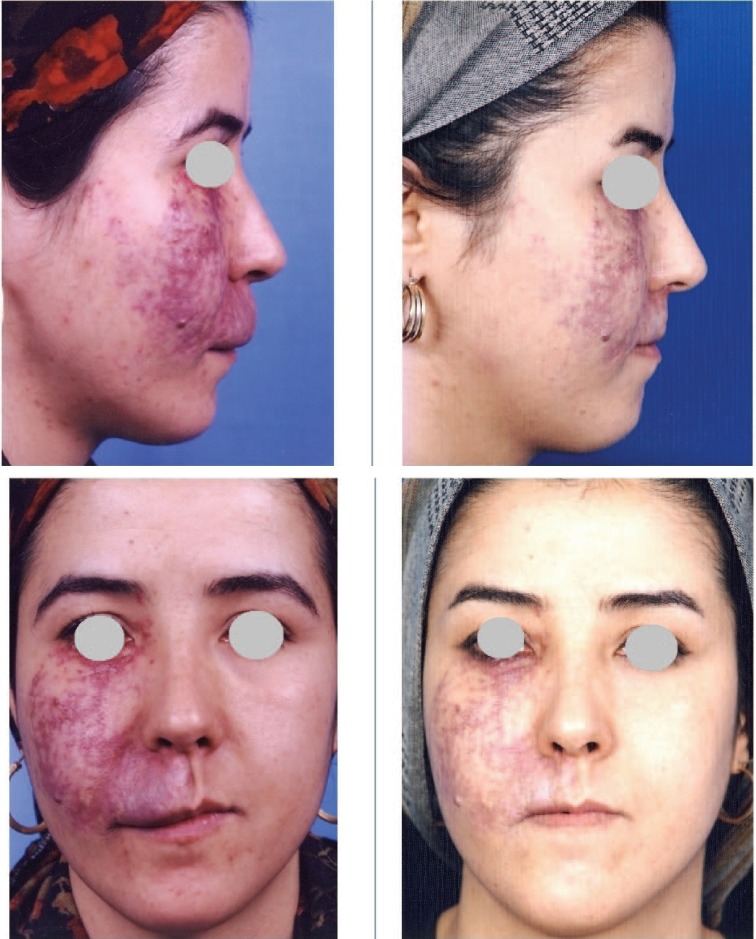
A 34 years old lady with mixed capillary and venous malformation of right side of mid face and right side of lip (left). She received 6 injections of the combination of bleomycin, triamcinolone and epinephrine. Three injections with 1 month interval, 2 injections with 2 months interval, 1 injection 6 months later. Post-injection photos 1 year later (Right).

**Fig. 4:**
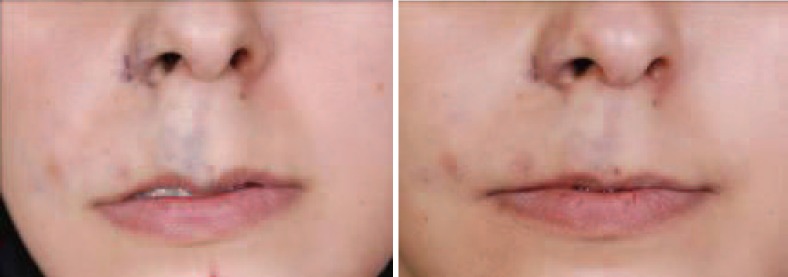
A 26 years old lady with venous malformation of right upper lip and alar base. She received 3 injections of the combination of bleomycin, triamcinolone and epinephrine with 2 months interval (Left) result after 6 months (Right).

**Fig. 5:**
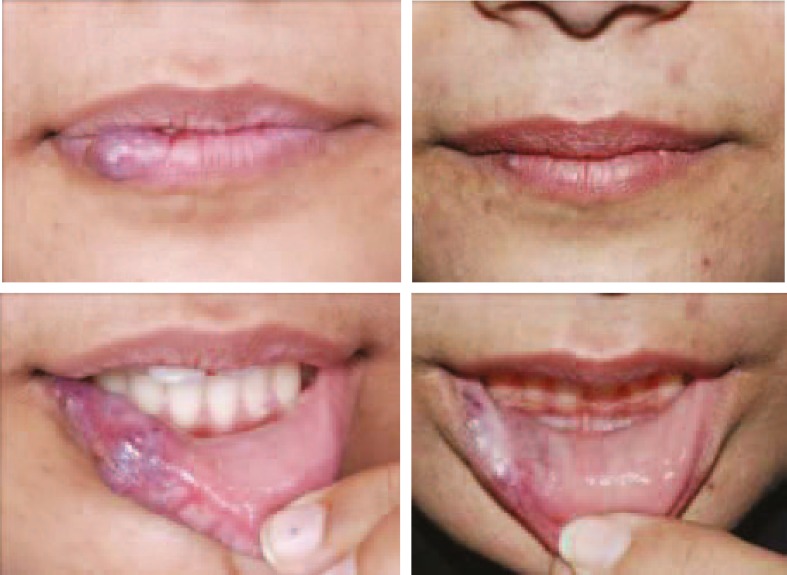
A 33 years old lady with small venous malformation of lip vermilion (left).She received 3 injections of the combination of bleomycin, triamcinolone and epinephrine with 3 months intervals. After 6 months the result is as shown (Right).

**Fig.6:**
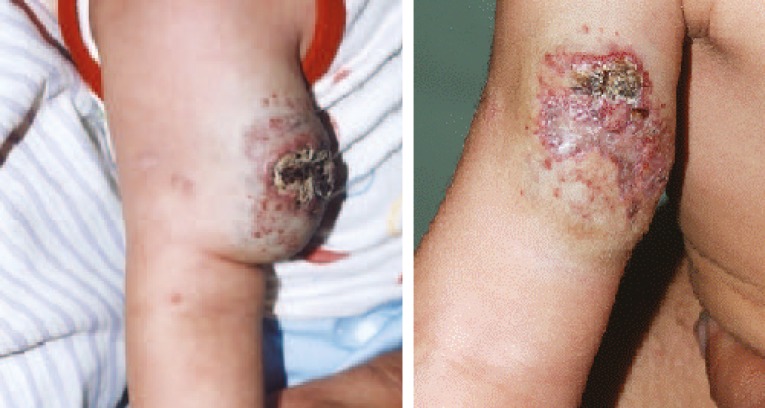
An eight months old girl with ulcerated hemangioma of right arm after two injections of combination therapy and results after five months.

Assessment of healing was based on visual evaluation of physicians in charge, identifying patient’s satisfaction (by using forms that divided patient’s satisfaction to four groups including poor, acceptable, good and excellent) and comparing serial photographs indicating reduction of lesion’s size, flow, dimension, asymmetry and change of color (Figures 1-6).

**Fig. 7:**
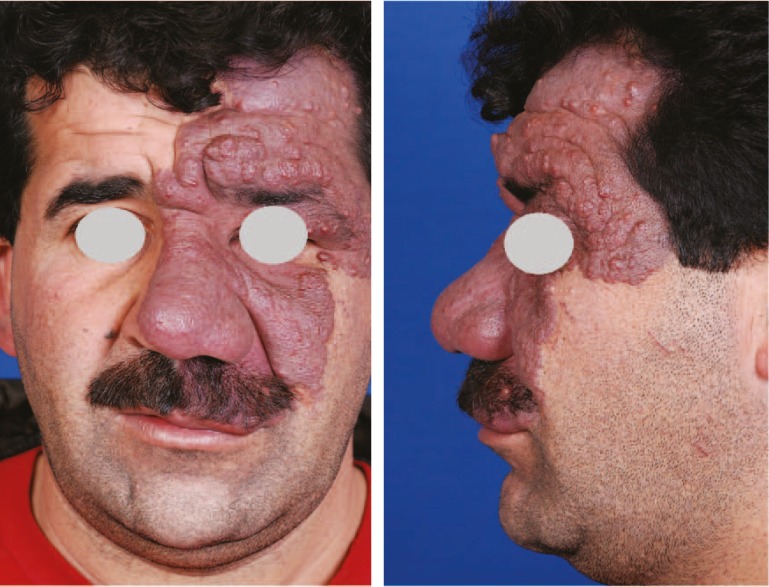
Case of port wine stain without any response to 3 injections of combination therapy.

By the end of the follow-up time, the success rate among hemangiomas and low-flow arteriovenous malformation and mixed lesions was excellent. On the other hand, port wine stains and high-flow arteriovenous malformation’ response to the injection was relatively poor. Two of the participants showed ulcers after injection in the central region of the primary lesion that healed in a week. One patient had hyper-pigmentation in the lesion zone after healing and two patients had hypopigmentation. Poor response to treatment was seen in 6 patients with port wine stains and highflow arteriovenous malformation (Figure 7).

## DISCUSSION

Bleomycin is an anti-tumor drug that is discovered in 1966.^12^ This drug influences the tissue in two ways: It inhibits DNA synthesis^13^ and also has sclerosing characteristics that affects vascular endothelium.^14^ It has been successfully treated hemangiomas while injected intralesionally, with a success rate of 87% and venous malformations with a success rate of 84%.^10^ This drug has low toxicity^15^ and it seems that bleomycin is a good treatment modality for vascular anomalies, but it has some complications (mainly localized) too. Muir *et al. *reported flu-like symptoms, ulceration, cellulitis and partial, temporary hair loss in some patients.^15^ Luce has reported pulmonary fibrosis in some oncologic patients who went under treatment with high intravenous dose of bleomycin^16^ in 1997. A study was performed that used bleomycin for treatment of painful, massive hemangiomas in children and reported that all cases relieved from pain and had a substantial reduction in the mass of lesion.^17^ Omidvari *et al. *assessed the effectiveness of intralesional injection of bleomycin with two weeks intervals for 4 to 6 times in treatment of complicated hemangiomas. They observed the maximum of lesion regression occurred in the first three months of treatment. They had a follow-up of 6 months and reported no recurrence or systemic and local complications. They concluded that bleomycin is a good therapeutic modality for treatment of hemangiomas especially in painful or massive lesions.^11^

The latest classification for vascular anomalies is approved by ISSVA (International Society for the Study of Vascular Anomalies) in Rome workshop in 1996. It has suggested two categories for vascular anomalies including hemangiomas and vascular malformation. Several methods and drugs have been applied in treatment of vascular anomalies, however there is no FDA-approved gold standard treatment method.^18^ Glucocorticosteroid therapy has been administrated in many cases as the first-line treatment, but its action mechanism is not yet well determined^18^ and it has a success rate of about 45%^19^ to 76%20 which is lower than bleomycin. Goyal *et al. *reported some cases of adrenal suppression in patients suffering from periocular hemangioma after being treated with local injection of steroids.^21^ Interferon-alpha is an anti-angiogenic agent that has been presented previously for the first time.^18^ It has treated 58% of hemangiomas, but it is expensive, needs long term treatment modality and its efficacy is uncertain.^17^,^22^ Laser treatment is another therapeutic method restricted to superficial layer of dermis and is not effective for lesions in deep layers of skin.^10^ Chen *et al. *treated arteriovenous malformations of maxillofacial region with a multidisciplinary approach using superselective intra-arterial embolization, bone wax packing of bone cavity and sclerotherapy. They achieved agreeable results after a mean of 13.5 months follow-up.^23^ Fathi *et al. *reported a case of arteriovenous malformation that they treated with a combination of treatment modality. The arteriovenous malformation was located in a dental socket and they applied preoperative embolization and external carotid artery ligation, dental extraction, curettage and packing.^24^ Hasan *et al. *compared *in vitro *effectiveness pattern of five common corticosteroids on angiogenesis of hemangiomas. They showed that dexamethasone and triamcinolone had similar effects from day 7 and they suggested that these two corticosteroids were proper for treatment of hemangiomas while effective treatment with methylprednisolone needs higher or repeated dose. They also claimed that hydrocortisone was not effective for treatment of hemangiomas.^25^

It seems that using the combination of bleomycin with a proper corticosteroid (whether dexamethasone or triamcinolone) may induce a faster healing. The low dosage of epinephrine helps vasoconstriction of local capillaries and improves the local effects of the bleomycin and triamcinolone. We suggest that later studies could compare the effectiveness of sole bleomycin, sole triamcinolone and the combination of bleomycin-triamcinolone treatment in order to identify the exact differences.

## CONFLICT OF INTEREST

The authors declare no conflict of interest.
